# Bond Strength of Composite Resin to Pulp Capping Biomaterials after Application of Three Different Bonding Systems

**DOI:** 10.5681/joddd.2013.024

**Published:** 2013-08-30

**Authors:** Zahra Jaberi-Ansari, Maryam Mahdilou, Maryam Ahmadyar, Saeed Asgary

**Affiliations:** ^1^Associate Professor, Department of Operative Dentistry, Faculty of Dentistry, Shahid Beheshti University of Medical Sciences, Tehran, Iran; ^2^Private Practice, Tehran, Iran; ^3^Researcher, Dental Research Center, Research Institute of Dental Sciences, Shahid Beheshti University of Medical Sciences, Tehran, Iran; ^4^Professor of Endodontics, Iranian Center for Endodontic Research, Research Institute of Dental Sciences, Shahid Beheshti University of Medical Sci-ences, Tehran, Iran

**Keywords:** Bonding agent, bond strength, calcium enriched mixture, composite resin, mineral trioxide aggregate

## Abstract

***Background and aims.*** Bonding of composite resin filling materials to pulp protecting agents produces an adhesive joint which is important for the quality of filling as well as success of restoration. We aimed to assess the bond strength of composite resin to three pulp capping biomaterials: Pro Root mineral trioxide aggregate (PMTA), Root MTA (RMTA) and calcium enriched mixture (CEM) cement, using three bonding systems [a total-etch (Single Bond) and two self-etch systems (Protect bond and SE Bond)].

***Materials and methods.*** Ninety acrylic molds, each containing a 6×2-mm hole, were divided into 3 groups and filled with PMTA, RMTA and CEM cements. The samples in each experimental group were then randomly divided into 3 sub-groups; Single Bond, Protect Bond and SE Bond bonding systems were applied to the tested materials. Cylindrical forms of composite resin (Z100, 2×2 mm) were placed onto the samples and cured. Shear bond strength values were measured for 9 subgroups using a universal testing machine. Data were analyzed using two-way ANOVA.

***Results.*** The average shear bond strengths of Z100 composite resin after application of Single Bond, Protect Bond and SE Bond systems were as follows; PMTA: 5.1±2.42, 4.56±1.96 and 4.52±1.7; RMTA: 4.71±1.77, 4.31±0.56 and 4.79±1.88; and CEM cement: 4.75±1.1, 4.54±1.59 and 4.64±1.78 MPa, respectively. The type of pulp capping material, bonding system and their interacting effects did not have a significant effect on the bond strengths of composite resin to pulp capping biomaterials.

***Conclusion.*** Within the limitations of this in vitrostudy, bond strength of composite resin to two types of MTA as well as CEM cement were similar following application of the total-etch or self-etch bonding systems.

## Introduction

In recent years, vital pulp therapy (VPT) has received considerable attention in dentistry, especially in endodontics.^1^ The aim of VPT is maintenance of the health and vitality of the dental pulp following traumatic injuries and carious pulp exposures.^2^ Historically, this treatment was carried out using calcium hydroxide but was not widely accepted due to unpredictable results.^3^ Introduction of novel dental biomaterials, supported by acceptable scientific evidence, has led to increased application of VPT technique in recent years.^1,4^

Mineral trioxide aggregate (MTA) has received considerable attention in VPT due to its proper biological characteristics and favorable histological/clinical results.^4,5^ MTA consists of hydrophilic particles which set in the presence of moisture by formation of calcium hydroxide and silicate hydrate gel. Grey and white ProRoot MTA (PMTA) have different chemical compositions,^6^ and other commercial forms of this biomaterial such as Root MTA (RMTA) have been manufactured in Iran. RMTA has demonstrated similar results with PMTA in terms of clinical/radiographic success of pulpotomy of primary molars as well as bacterial and dye microleakage.^7,8^ It has been recommended that MTA should be used in cases requiring VPT, in apical barrier placement for necrotic teeth with open apices, in perforation repair and in root-end fillings;^9^ on the other hand, MTA has some known drawbacks such as a long setting time, high cost and discoloration potential.^10^

Calcium enriched mixture (CEM) cement is a novel endodontic biomaterial that contains various calcium combinations with numerous clinical applications such as root-end filling,^11^ apexogenesis,^12^ permanent molar pulpotomy,^13^ perforation repair,^14^ treatment of inflammatory root resorption^15^ and regenerative endodontics.^16^ This biomaterial contains some superior physical characteristics compared to MTA; such as flow, film thickness and shorter setting time.^17^ It has been recently reported that using CEM cement for direct pulp capping of a molar tooth with irreversible pulpitis and apical periodontitis has shown favorable results.^18^

To date, a limited number of studies have been carried out on the bond strength between PMTA and restorative materials using various bonding systems.^19,20^ A recent study assessed the bond strength of PMTA and CEM cement to composite resin with and without the use of acid etching.^21^ However, the effect of various bonding systems on RMTA and CEM cement has not been investigated yet. The aim of the present study was to investigate the bond strength of composite resin (Z100) to three different pulp capping biomaterials (CEM cement, RMTA, and PMTA) using three different bonding systems: a total-etch (Single Bond) and two self-etch systems (Protect Bond and SE Bond).

## Materials and Methods

This was a laboratory experimental study. Ninety acrylic blocks were prepared using quick-setting acrylic resin (Acropars, Tehran, Iran) from a cast metal mold measuring 2 × 2 cm. Each of the acrylic blocks contained a central hole measuring 6 mm in diameter and 2 mm in depth.^19^ The molds were divided into three experimental groups. In group 1, PMTA (ProRoot, Dentsply, OK, USA) was prepared according to manufacturer's instructions, placed into the holes using a spatula and pressed using a glass slab. MTA was then covered with a damp cotton pellet and stored in an incubator (Peco, Tehran, Iran) at 95% humidity and 37°C for 48 hours in order to allow complete hardening of the materials. In groups 2 and 3, the samples were filled with RMTA (Salamifar, Tehran, Iran) and CEM cement (Bionique Dent, Tehran, Iran), respectively. The samples in three experimental groups were randomly divided into three subgroups of 10 each (totally 9 subgroups).

In the single bond subgroup, the surface of the biomaterial was etched using an acid phosphoric gel for 15 seconds (Super Etch GEL, CE 37.5%), rinsed with water for 10 seconds and excess moisture was absorbed using paper points in order to leave a relatively dry surface. Two consecutive layers of bonding material (Single Bond, 3M ESPE, USA) were then applied onto the surface. In the Protect Bond subgroup, Clearfil (Kurary, Okayama, Japan) was used. The primer was initially applied to the samples and left for 20 seconds. Gentle air was used from a 3-in-1 syringe held from a distance of 2 cm to evaporate the solvent and the bonding agent was applied. In the self etch bond subgroup, Clearfil SE Bond bonding agent (Kuraray, Okayama, Japan) was applied to the surface of the biomaterials, followed by application of the primer which was left for 20 seconds. Gentle air was used from a 3-in-1 syringe held from a distance of 2 cm to evaporate the solvent. The bonding agent was then applied.

Following application of the bonding agent in all of the three groups, a 3-in-1 syringe was used to gently dry the surface for 5 seconds from a distance of 2 cm. The surface was then light-cured using a diode machine (Demetoron LC, SDS Kerr, USA) at a light intensity of 1200 mW/cm^2^ for 10 seconds. Composite resin (Z100, 3M ESPE, USA) was then placed in transparent plastic matrix cylinders (2 mm in diameter and 2 mm in height) and light-cured for 40 seconds. The prepared samples were stored in an incubator for 24 hours with 95% humidity and 37°C temperature. The shear bond strength of the samples was measured using a universal testing machine (Zwick/Roell 2020, Germany). A force of 1.0 mm/min was applied to the samples using the knife-edge blade of the machine. Shear bond strength was measured in MPa by dividing the highest amount of force to the surface area of the samples.

Two-way ANOVA was used for investigation of the effect of the variables on various bond strength values.

## Results

The mean and standard deviation values for shear bond strength of composite resin to the biomaterials are presented in Table 1. The results of two-way ANOVA revealed that the type of biomaterial (P=0.98), bonding system (P=0.46) and the relative effect of the bonding system and capping material (P=0.76) did not have a statistically significant effect on the shear bond strength of composite resin. Considering the lack of statistical significance in assessing overall differences between the groups, comparison of pairs of groups was not carried out.

**Table 1:** Statistical indices for shear bond strength of z100 composite resin to the tested biomaterials

Group / Bonding System (n = 10)	Mean	SD	Min	Max
PMTA				
Single Bond	5.10	2.42	2.49	9.56
Protect Bond	4.56	1.96	2.39	7.57
SE Bond	4.52	1.70	2.57	7.71
RMTA				
Single Bond	4.71	1.77	2.61	7.76
Protect Bond	4.31	0.56	2.38	7.36
SE Bond	4.79	1.88	2.84	8.42
CEM				
Single Bond	4.75	1.10	2.31	6.11
Protect Bond	4.54	1.59	2.38	7.23
SE Bond	4.64	1.78	2.51	7.93

## Discussion

Placement of a restoration immediately after VPT is recommended in order to create and maintain an effective coronal seal which is essential for success of VPT. Composite resin is recommended due to lower forces placed onto the pulp capping biomaterial during placement of the permanent restoration. The test for shear bond strength is reliable for experimental evaluation of various dental materials under laboratory circumstances.^22^ In this study, the bond strength values of the new generation of pulp capping biomaterials to composite resin (Z100) were assessed using three different bonding systems; there were no significant differences between either of the groups/subgroups.

The bond strength of pulp capping biomaterials to composite resin depends on their physical and chemical properties. MTA and CEM are hydrophilic biomaterials which harden in the presence of moisture. The main ingredients of gray or white MTA include calcium oxide, silica and bismuth oxide;^6^ on the other hand, one of the main ingredients of CEM cement is phosphorous which is present in negligible amounts in MTA.^23^ Over both biomaterials, hydroxyapatite crystals are precipitated in phosphate-buffered solution.^24^ Although the particle sizes of these biomaterials are different, the pH and setting/working times are similar.^17^ In a study by Oskoee et al,^21^ there was no reported difference between shear bond strength of adhesive resin cylinders to MTA and CEM cement using Single Bond. The researchers assessed the shear bond strength of composite resin to MTA and CEM cement with and without the use of acid etch; it was concluded that etching did not affect the bond strength, and that surface preparation of the above biomaterials was not necessary in VPT. The results of the above study confirmed our results regarding shear bond strength values using Single Bond adhesive system in the etched samples.

Due to the possible inadvertent effects on setting time and risk of dislodging/dissolving of MTA following acid etching/irrigation, Shokouhinejad et al^25^ did not recommend placement of composite resin on fresh MTA. A scanning electron microscope evaluation of the effects of acid etching on surface characteristics of MTA showed that the disordered structure and spindle shaped crystals are removed during this process;^26^ thus the selective removal of the matrix around the crystals leads to a sponge-like surface that is suitable for bonding to composite resins without significantly affecting the structure of MTA/CEM cement. Self-etching bonding systems contain acidic and hydrophilic monomers which do not require irrigation after etching. With a reduction in working time and the number of stages, the application of these systems has become simpler and less technique-sensitive.^27^ In this study, we compared the bond strength of self-etch bonding system, which does not require separate etching/irrigation stages, with the single-bond system, which requires a separate etching stage; it was shown that there were no significant differences between the three pulp capping biomaterials.

The most important step of successful vital pulp therapy is placing a biomaterial over the pulp as well as a perfect restoration to achieve an immediate hermetic coronal seal and to minimize further microbial leakage/irritation; hence, we suggest that in future studies experiments be carried out on fresh pulp capping biomaterials.

## Conclusion

It can be concluded that the shear bond strength values of composite resin (Z100) to CEM cement and both MTAs were similar following application of the three bonding systems; therefore, composite resin restorations can be directly bonded to the above biomaterials.

## Acknowledgements

The authors would like to thank the Dental Research Center of Shahid Beheshti University of Medical Sciences for provision of resources for this study which was carried out as a student thesis (number 3038).

## References

[1] Aguilar P, Linsuwanont P (2011). Vital pulp therapy in vital permanent teeth with cariously exposed pulp: a systematic review. J Endod.

[2] Trope M (2008). Regenerative potential of dental pulp. Pediatr Dent.

[3] Tziafas D, Pantelidou O, Alvanou A, Belibasakis G, Papadimitriou S (2002). The dentinogenic effect of mineral trioxide aggregate (MTA) in short-term capping experiments. Int Endod J.

[4] Asgary S, Eghbal MJ (2013). Treatment outcomes of pulpotomy in permanent molars with irreversible pulpitis using biomaterials: A multi-center randomized controlled trial. Acta Odontol Scand.

[5] Eghbal MJ, Asgary S, Baglue RA, Parirokh M, Ghoddusi J (2009). MTA pulpotomy of human permanent molars with irreversible pulpitis. Aust Endod J.

[6] Asgary S, Parirokh M, Eghbal MJ, Brink F (2005). Chemical differences between white and gray mineral trioxide aggregate. J Endod.

[7] Haghgoo R, Abbasi F (2010). Clinical and Radiographic Success of Pulpotomy with MTA in Primary Molars: 30 Months Follow up. Iran Endod J.

[8] Kazem M, Eghbal MJ, Asgary S (2010). Comparison of bacterial and dye microleakage of different root-end filling materials. Iran Endod J.

[9] Torabinejad M, Chivian N (1999). Clinical applications of mineral trioxide aggregate. J Endod.

[10] Parirokh M, Torabinejad M (2010). Mineral trioxide aggregate: a comprehensive literature review--Part III: Clinical applications, drawbacks, and mechanism of action. J Endod.

[11] Asgary S, Eghbal MJ, Ehsani S (2010). Periradicular regeneration after endodontic surgery with calcium-enriched mixture cement in dogs. J Endod.

[12] Nosrat A, Asgary S (2010). Apexogenesis treatment with a new endodontic cement: a case report. J Endod.

[13] Asgary S, Eghbal MJ, Ghoddusi J, Yazdani S (2013). One-year results of vital pulp therapy in permanent molars with irreversible pulpitis: an ongoing multicenter, randomized, non-inferiority clinical trial. Clin Oral Investig.

[14] Samiee M, Eghbal MJ, Parirokh M, Abbas FM, Asgary S (2010). Repair of furcal perforation using a new endodontic cement. Clin Oral Investig.

[15] Asgary S, Nosrat A, Seifi A (2011). Management of inflammatory external root resorption by using calcium-enriched mixture cement: a case report. J Endod.

[16] Nosrat A, Seifi A, Asgary S (2011). Regenerative endodontic treatment (revascularization) for necrotic immature permanent molars: a review and report of two cases with a new biomaterial. J Endod.

[17] Asgary S, Shahabi S, Jafarzadeh T, Amini S, Kheirieh S (2008). The properties of a new endodontic material. J Endod.

[18] Asgary S, Nosrat A, Homayounfar N (2012). Periapical Healing After Direct Pulp Capping With Calcium-enriched Mixture Cement: A Case Report. Oper Dent.

[19] Tunc ES, Sonmez IS, Bayrak S, Egilmez T (2008). The evaluation of bond strength of a composite and a compomer to white mineral trioxide aggregate with two different bonding systems. J Endod.

[20] Neelakantan P, Grotra D, Subbarao CV, Garcia-Godoy F (2012). The shear bond strength of resin-based composite to white mineral trioxide aggregate. J Am Dent Assoc.

[21] Oskoee SS, Kimyai S, Bahari M (2011). Comparison of shear bond strength of calcium-enriched mixture cement and mineral trioxide aggregate to composite resin. J Contemp Dent Pract.

[22] Perdigao J, Lopes M (1999). Dentin bonding--questions for the new millennium. J Adhes Dent.

[23] Asgary S, Eghbal MJ, Parirokh M (2009). Comparison of mineral trioxide aggregate's composition with Portland cements and a new endodontic cement. J Endod.

[24] Asgary S, Eghbal MJ, Parirokh M, Ghoddusi J (2009). Effect of two storage solutions on surface topography of two root-end fillings. Aust Endod J.

[25] Shokouhinejad N, Nekoofar MH, Iravani A, Kharrazifard MJ, Dummer PM (2010). Effect of acidic environment on the push-out bond strength of mineral trioxide aggregate. J Endod.

[26] Kayahan MB, Nekoofar MH, Kazandag M (2009). Effect of acid-etching procedure on selected physical properties of mineral trioxide aggregate. Int Endod J.

[27] Bishara SE, VonWald L, Laffoon JF, Warren JJ (2001). Effect of a self-etch primer/adhesive on the shear bond strength of orthodontic brackets. Am J Orthod Dentofacial Orthop.

